# Interactions of Analgesics with Cisplatin: Modulation of Anticancer Efficacy and Potential Organ Toxicity

**DOI:** 10.3390/medicina58010046

**Published:** 2021-12-28

**Authors:** Azza El-Sheikh, Zenat Khired

**Affiliations:** 1 Basic Health Sciences Department, Faculty of Medicine, Princess Nourah Bint Abdulrahman University, Riyadh 11671, Saudi Arabia; 2 Department of Pharmacology, Faculty of Medicine, Minia University, El-Minia 61511, Egypt; 3 Department of Surgery, Faculty of Medicine, Jazan University, Jazan 45142, Saudi Arabia; zkherd@jazanu.edu.sa

**Keywords:** cisplatin, analgesics, acetaminophen, non-steroidal anti-inflammatory drugs, morphine, cytotoxicity

## Abstract

Cisplatin (CDDP), one of the most eminent cancer chemotherapeutic agents, has been successfully used to treat more than half of all known cancers worldwide. Despite its effectiveness, CDDP might cause severe toxic adverse effects on multiple body organs during cancer chemotherapy, including the kidneys, heart, liver, gastrointestinal tract, and auditory system, as well as peripheral nerves causing severely painful neuropathy. The latter, among other pains patients feel during chemotherapy, is an indication for the use of analgesics during treatment with CDDP. Different types of analgesics, such as acetaminophen, non-steroidal anti-inflammatory drugs (NSAIDS), and narcotic analgesics, could be used according to the severity of pain. Administered analgesics might modulate CDDP’s efficacy as an anticancer drug. NSAIDS, on one hand, might have cytotoxic effects on their own and few of them can potentiate CDDP’s anticancer effects via inhibiting the CDDP-induced cyclooxygenase (COX) enzyme, or through COX-independent mechanisms. On the other hand, some narcotic analgesics might ameliorate CDDP’s anti-neoplastic effects, causing chemotherapy to fail. Concerning safety, some analgesics share the same adverse effects on normal tissues as CDDP, augmenting its potentially hazardous effects on organ impairment. This article offers an overview of the reported literature on the interactions between analgesics and CDDP, paying special attention to possible mechanisms that modulate CDDP’s cytotoxic efficacy and potential adverse reactions.

## 1. Introduction

Cisplatin (CDDP) is a platinum-based agent that has long been used in the treatment of various types of malignancies [1]. Unfortunately, CDDP may cause toxic side effects on normal human tissues that might lead to multiple organ damage [2,3]. During chemotherapy, several patients may suffer from pain and are likely to take medications to relief it. According to the level of the pain, these medications may range from acetaminophen or non-steroidal anti-inflammatory drugs (NSAIDs), in the case of mild to moderate pain, reaching up to stronger pain killers such as narcotic analgesics in the case of severe pain. It is possible that administering these medications concomitantly with CDDP might augment CDDP-induced organ toxicity or alter its anticancer efficacy. To date, the interactions of analgesics with CDDP have not been fully reviewed. Here, data were collected from the literature to formulate an updated review of the molecular mechanisms that might be involved in the interactions of different types of analgesics with CDDP, and the implications of such interactions on rational use of analgesics for the treatment of pain during CDDP cancer chemotherapy in humans.

## 2. CDDP Efficacy and Toxicity

**Figure 1:**
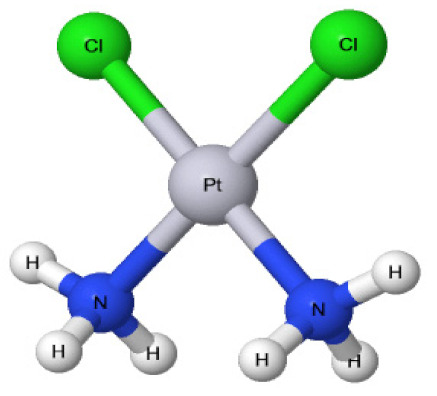
Chemical structure of cisplatin. Two neutral ammonia (NH_3_) ligands and two chloride (Cl) anions are coordinated to the central platinum (Pt) ion.

CDDP was originally created by M. Peyrone in 1844, and in 1893 its chemical composition (Figure 1) was first revealed by Alfred Werner [4]. This was followed by the accidental discovery of CDDP’s cytotoxic actions by Dr. Rosenborg in 1965 [5] and the approval of CDDP for medical use in 1978 [6]. Since then, this anticancer drug has been used to treat numerous neoplasms, including those of the testes, ovaries, uterus, breasts, bladder, gastrointestinal tract, lung, bone, and brain [4,6,7]. CDDP performs its anticancer actions through forming covalent intra-strand DNA adducts between its CDDP platinum complexes and the neoplastic cell DNA, which causes subsequent DNA damage and obstruction of efficient DNA repair, resulting in restriction of DNA synthesis and inhibition of tumor cell growth [8,9]. CDDP induces free-radical formation, especially reactive oxygen species (ROS) that can be the initial trigger of cancer-programmed cellular death: apoptosis. This is due to the induction of pro-apoptotic factors, such as Bax and Bid, and the dysregulation of anti-apoptotic factors, such as Bcl-2, as well as the activation of caspases, which result in an apoptotic cascade [10]. Unfortunately, CDDP, by the same mechanisms, may also affect normal tissues, resulting in morbid, and sometimes fatal, side effects. Nearly a quarter of patients treated with CDDP develop nephrotoxicity as a side effect, through epigenetic DNA methylation, histone modification, oxidative stress, inflammation, and apoptosis [11]. Similar mechanisms are involved in CDDP-induced hepatotoxicity [12,13], cardiotoxicity [14], gastrointestinal toxicity [15], and ototoxicity [16]. CDDP was also found to be neurotoxic [17], affecting mainly sensory nerves, inducing painful neuropathy as a side effect [18], which may be a strong indication for the use of analgesics concomitantly with CDDP to relief such pain. 

## 3. Interactions of Acetaminophen with CDDP

Acetaminophen, also acknowledged as paracetamol, is a para-aminophenol derivative that may be used for the management of mild to moderate pain during CDDP anticancer treatment, as well as for treatment of CDDP-chemotherapy-related fever [19]. Since it lacks anti-inflammatory properties, acetaminophen is usually not considered as one of the NSAIDs. It was reported that acetaminophen may act as a chemo-enhancer that promotes the cytotoxic effect of CDDP on hepatocarcinoma and hepatoblastoma cells, by decreasing GSH and the induction of oxidative stress [20]. The same mechanism was seen when an acetaminophen/CDDP combination was administered to resistant atypical teratoid rhabdoid pediatric tumor cells [21] and human ovarian carcinoma [22]. Unfortunately, both acetaminophen and CDDP are considered hepato- and nephrotoxic [23], thus may be cautiously used concomitantly if the patient has kidney or liver function impairment.

## 4. Interactions of NSAIDs with CDDP

**Figure 2:**
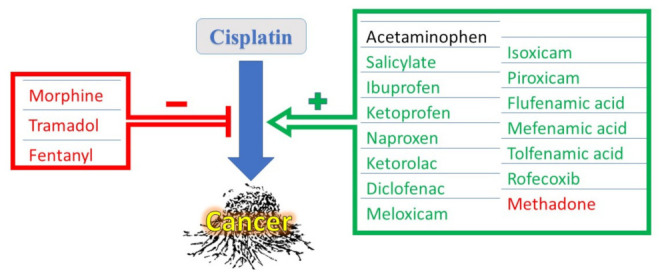
Effect of different analgesics on cisplatin’s anticancer efficacy. Analgesic names in green letters are non-steroidal anti-inflammatory drugs and those in red letters are narcotic analgesics.

The major mechanism of action of NSAIDs in treating pain is through the inhibition of the cyclooxygenase (COX) enzyme that catalyzes the formation of eicosanoids that mediate inflammation and pain, such as thromboxanes, prostaglandins, and prostacyclins, from membrane phospholipid arachidonic acid [24]. Since inflammation offers a suitable microenvironment for malignancies to develop, it is conceivable that NSAIDs possessing anti-inflammatory properties may help in the management of cancer. Interestingly, CDDP can induce COX-2 that causes the secretion of large amounts of prostaglandins, resulting in a decrease in CDDP chemotherapeutic efficacy [25,26]. It is, thus, logical that NSAIDs, especially selective COX-2 inhibitors, might act as chemosensitizers to resistant cancers, making them more susceptible to treatment by CDDP [27]. Interestingly, several non-selective NSAIDs, such as ketoprofen and naproxen, were assumed to have cytotoxic, anti-proliferative effects on their own, which was independent from the COX pathway, but seemed to be, at least partially, due to the induction of the NSAID-activated gene; NAG-1 [28]. NSAIDs that hold some potential to improve CDDP anticancer effects are summarized in Figure 2.

The NSAIDs can be subdivided into salicylates, propionic acids, acetic acids, enolic acids, anthranilic acids, naphthylalanine, and selective COX-2 inhibitors [24]. Due to their chemical diversity, NSAIDs show different levels of selectivity on inhibiting COX-1 and COX-2 enzymes [29]. In general, most non-selective NSAIDs are known to induce gastric ulceration [30], as well as having renal side effects including tubulointerstitial nephritis, nephrotic syndrome, acute kidney injury, and chronic kidney disease [31], whereas COX-2 selective NSAIDs may cause cardiovascular side effects [32]. Still, there are several exceptions. For example, the non-selective NSAID loxoprofen, might not harm the gastric mucosa as much as its peer NSAIDs [33]. Its derivative fluoro-loxoprofen, might even have gastroprotective effects [34]. Indomethacin, on the other hand, was reported to have the highest gastrotoxic potential [35].

### 4.1. Interactions of Salicylates with CDDP

Salicylate, the prototype of NSAIDs, has shown promising anticancer effects [36,37,38]. Several studies indicated that salicylate can, by different mechanisms, increase the cytotoxic efficacy of CDDP. In one study, salicylate was reported to improve the anti-tumor effects of CDDP against T cell lymphoma via changing the tumor microenvironment pH, altering the expression of the cell cycle’s regulatory/apoptotic factors, such as p53, bcl-2, bcl-xL, cyclin B1, and D, as well as cytokines IFN-γ, VEGF, IL-4, and -10 [39]. Other studies showed that salicylate also increased the anti-tumor effect of CDDP against osteosarcoma, through modulating the NF-κB pathway [40], and against non-small cell lung carcinoma stem-like cells by repressing migration through acting on the mTOR-Akt axis [41]. In addition, salicylate improved CDDP toxicity against colon cancer cells through preventing NF-κB binding to a COX-2 promoter [42] against lung cancer cells, by abrogating cancer cell stemness [43], against epithelial ovarian cancer cells by increasing p53 acetylation and promoting apoptosis [44], and against oesophageal squamous cell carcinoma through epigenetic modulation of chromatin by altering histone acetylation levels [45]. Due to these beneficial effects, asplatin or prodrug platin-A, which are CDDP-based Pt(IV) prodrugs complexed with salicylate, were developed to improve cytotoxicity against resistant cancer cells [46,47]. Despite its obvious potentiating cytotoxic effects on tumor cells, salicylate might have a protective effect on normal cells against CDDP-induced nephrotoxicity, ototoxicity, and neurotoxicity [48,49,50].

### 4.2. Interaction of Propionic Acid-Derived NSAIDs (Profens) with CDDP

Ibuprofen, one of the propionic acid-derived NSAIDs, showed cytotoxic effects when administered alone to human promyelocytic leukemia and colon carcinoma cells [51]. Some studies succeeded in synthesizing lipid encapsulated ibuprofen metallodrug nanoparticles to overcome CDDP chemoresistance in glioblastoma cancer cells [52]. It was also reported that ibuprofen increased CDDP anticancer efficacy against lung cancer cells through depletion of heat shock protein 70, thus enhancing tumor cell apoptosis [53]. In addition, combining ibuprofen with CDDP caused a higher cytotoxic effect on thyroid and pancreatic cancer cells in vitro [54]. Furthermore, an ibuprofen/CDDP combination reversed CDDP resistance in non-small-cell lung cancer through a COX-independent mechanism [55]. In addition to increasing CDDP’s cytotoxic efficacy, ibuprofen was reported to inhibit human ovarian cancer cell metastasis into several organs, such as the liver, lungs, bone marrow, and spleen in mice [56]. Unfortunately, through stimulating oxidative stress, ibuprofen might cause toxicity similar to CDDP on the kidneys and liver [57,58].

Ketoprofen, another propionic acid-derived NSAID, was conjugated with CDDP-based Pt(IV) prodrug to form ketoplatin that could delay breast cancer cells’ tumor growth and had less systemic toxic effects compared to CDDP alone in vitro and in vivo [59]. Interestingly, ketoprofen was suggested to protect against CDDP-induced nephrotoxicity [60], which is in line with more recent findings that ketoprofen has no nephrotoxic effects [61]. Several trials were also performed to assess the anti-tumor effects of combining CDDP with a third propionic acid-derived NSAID, naproxen [62,63], where the combination showed higher cytoxicity than CDDP alone on human cancer cells of the lungs and ovaries, with less toxicity on normal human liver cells [64]. Similar results were shown for a naproxen/CDDP combination on triple-negative breast cancer [65], as well as on ovarian endometrioid adenocarcinoma, lung adenocarcinoma, malignant pleural mesothelioma, and colon carcinoma cells [28]. Carprofen alone was able to ameliorate canine osteosarcoma in vitro [66]. Novel NSAIDs were created, such as derivatives of naproxen, flurbiprofen, and ibuprofen, that showed promising anticancer effect against cultured human glioblastoma cells [67], as well as human liver, breast, and colon carcinoma cells [68]. Whether the anticancer effects of these NSAIDS would be additive to that of CDDP or not still needs further investigation.

### 4.3. Interaction of Acetic Acid-Derived NSAIDS with CDDP

One of the acetic acid-derived NSAIDs, indomethacin, attenuated the growth of human oesophageal squamous carcinoma cells [69]. Sulindac could also ameliorate the growth rate of oral tumor cells and help their elimination by natural killer cells [70]. In addition, sulindac could prevent the progression of colorectal cancer clinically, by up-regulating cyclin G2 which resulted in delaying tumor cell cycle progression [71]. Interestingly, sulindac showed comparable cytotoxic effects to those of CDDP when tested on HEK293 cells [72]. Given together with CDDP, ketorolac succeeded in reversing CDDP chemo-resistance in a patient-derived cell xenograft model [73]. Diclofenac also showed improved CDDP anticancer effects against human lung adenocarcinoma CDDP-resistant cells [74,75]. To the contrary to what is expected from non-selective COX inhibitors, diclofenac did not deteriorate CDDP-induced nephrotoxicity [74]. Nevertheless, diclofenac, as with CDDP, had the hazard of causing hepatotoxicity as an adverse effect [76].

### 4.4. Interaction of Enolic Acid Derivatives of NSAIDs (Oxicams) with CDDP

Meloxicam, an enolic acid derivative of NSAIDs with relative preferential selectivity to inhibit COX-2, had a synergistic effect on CDDP cytotoxicity in human osteosarcoma cells [77]. Interestingly, meloxicam protected the kidney from CDDP-induced renal lesions in mice [78]. Oxicams have been suggested as chemosensitizers of CDDP, and some trials attempted to develop CDDP–oxicam complexes as anticancer drugs, using meloxicam and isoxicam, where the results showed promising cytotoxic effects on different cell lines in vitro [79]. Piroxicam, another enolic acid derivative of NSAIDs, when given as an adjuvant to CDDP-loaded nanoparticles, increased apoptosis in mesothelioma cells [80]. Unfortunately, unlike meloxicam, piroxicam was shown to worsen CDDP-induced nephrotoxicity in rats [81]. Tenoxicam alone seemed tolerable in patients with renal impairment [82], but was reported to have an injurious effect on the liver [83].

### 4.5. Interaction of Anthranilic Acid and Naphthylalanine Derivatives of NSAIDs (Fenamates) with CDDP

The anthranilic acid derivatives, flufenamic and mefenamic acids, were reported to augment CDDP’s anticancer effect in vitro through inhibiting aldo–keto reductase 1C enzyme [84,85]. Similarly, tolfenamic acid was coupled with CDDP to form a nanoprodrug that had tumor apoptotic and anti-metastatic effects on breast cancer in vitro and in vivo [26]. On their own, neither meclofenamic nor niflumic acid showed promising anticancer effects against uterine cervical cancer and breast adenocarcinoma cells, respectively [86,87]. Concerning safety, meclofenamic acid could aggravate CDDP-induced renal damage [88]. However, meclofenamic acid seemed to have the potential to protect against CDDP-induced ototoxicity via improving the viability of ear hair cells [89]. Nabumetone, a naphthylalanine derivative, had an antiproliferative effect on MCF-7 and MDA-MB-231 breast carcinoma cells [90], with low toxic effects on gastric mucosa cells [91].

### 4.6. Interaction of COX-II Selective NSAIDS (Coxibs) with CDDP

Selective COX-2 inhibitors, frequently referred to as “coxibs”, were reported to have, on their own, promising potential for preventing and treating malignancies [92,93]. Administered with CDDP, rofecoxib was reported to enhance cytotoxic effects on gastric cancer cells by down regulating multidrug resistance protein 1 expression [94]. Nevertheless, combining CDDP with celecoxib did not improve the anticancer activity of CDDP against human esophageal squamous cell carcinoma xenograft model in vivo [95]. Despite their safety with regards to gastric ulceration, selective COX-2 inhibitors were reported to mediate cardiotoxicity [32]. Indeed, several members of this group, such as valdecoxib and rofecoxib, were removed from the market due to their potential cardiovascular hazards [96,97]. Despite its reported hazard on cardiomyocytes [98], celecoxib only received a box warning on its pack, but is still sold in the market. Interestingly, parecoxib was reported to have a protective effect on ischemia-reperfusion injury of the heart in rats [99]. Celecoxib showed protective effect against CDDP-induced nephrotoxicity [100]. Another coxib, still present on the market, etoricoxib, was tested for possible nephroprotective effects against CDDP-induced renal toxicity in rats, but, unfortunately, the results were not conclusive [101].

## 5. Interaction of Narcotic Analgesics with CDDP

Opioids have different impacts on cancer viability. Both morphine and fentanyl might promote cancer, while buprenorphine had no effect on cancer, and tramadol might ameliorate cancer by modulating the activity of natural killer cells [102]. Tramadol initiated apoptotic effects in colon cancer stem cells [103]. Still, tramadol might interfere with CDDP cytotoxicity via a different mechanism, as it suppresses gap junction activity [104]. It seems that opioids, especially μ- and κ-receptor agonists, suppressed natural killer cells cytotoxicity, promoting viability of cancer cells [105]. Indeed, fentanyl decreased the sensitivity of lung cancer cells to CDDP [106]. We have shown that morphine, the prototype agonist of opioid μ-receptor, also reduced the anticancer efficacy of CDDP on breast cancer cells [107]. An exception to this is methadone, another opioid μ-receptor agonist, that might enhance CDDP anticancer effects against bladder cancer [108], as well as head and neck cancer cells [109]. Regarding toxicity, we have previously reported the hazardous effects of morphine on CDDP-induced cardiotoxicity and hepatotoxicity [13,107]. Tapentadol was also reported to cause lung, heart, and neuronal toxicity [110], as well as hepatorenal toxic effects [111]. Further studies are needed to validate if tapentadol’s side effects would be cumulative to that of CDDP if taken together. Table 1 summarizes the effect of different analgesics on CDDP-induced toxicities.

## 6. Conclusions

Despite the absolute need for analgesics for the treatment of pain during cancer chemotherapy with CDDP, physicians should bear in mind the consequences of the combination of different analgesics on CDDP efficacy and toxicity. Rational evidence-based combinatorial therapy with CDDP and analgesics can provide enormous benefits in providing higher selectivity in targeting cancer cells and avoiding augmentation of the hazards of CDDP’s side effects. Still, it should be noted that the majority of available data concerning the interaction between CDDP and analgesics on the level of efficacy and toxicity were generally interpreted from in vitro or in vivo animal models. Future clinical studies are needed to verify the impact of the CDDP/analgesic interaction during actual patient chemotherapeutic settings.

## Figures and Tables

**Table 1:** Effect of analgesics on organ toxicity that may deteriorate or protect against cisplatin-induced organ/tissue damage.

Name of NSAID	Organ/Tissue	Effect	Type of Experiment	Ref.
Acetaminophen	Kidney	Nephrotoxicity	Animal study (rat)	[23]
	Liver	Hepatotoxicity		
NSAIDs ^1^	Kidney	Nephro-protective	Animal study (rat)	[48]
1. Salicylate	Auditory system	Protect against ototoxicity	Human study	[49]
	Neurons	Neuro-protective	In vitro	[50]
2. Propionic acid-derived NSAIDs				
Fluoro-loxoprofen	Stomach	Gastroprotective	Animal study (rats)	[34]
Ibuprofen	Kidney	Nephrotoxicity	Animal study (rat)	[57]
	Liver cells	Hepatotoxicity	In vitro	[58]
Ketoprofen	Kidney	Nephro-protective	Animal studies (rat and pig)	[60,61]
3. Acetic acid-derived NSAIDS				
Indomethacin	Stomach cells	Gastric ulceration	In vitro	[35]
Diclofenac	Kidney	Nephrotoxicity	Human (review)	[31]
	Liver	Hepatotoxicity	Animal study (rat)	[76]
4. Enolic acid-derived NSAIDs				
Meloxicam	Kidney	Nephroprotective	Animal study (mouse)	[78]
Piroxicam	Stomach	Gastric ulceration	Human (review)	[30]
	Kidney	Nephrotoxicity	Animal study (rat)	[81]
Tenoxicam	Liver	Hepatotoxicity	Animal study (rat)	[83]
5. Anthranilic acid-derived NSAIDs				
Meclofenamic acid	Kidney	Nephrotoxicity	Animal study (mouse) and in vitro	[88]
	Cochlear hair cell	Protect against ototoxicity	In vitro	[89]
6. COX-II ^2^ selective NSAIDS				
Valdecoxib	Heart	Cardiotoxicity	Human (review)	[96]
Rofecoxib	Heart	Cardiotoxicity	Animal study (rat)	[97]
Celecoxib	Cardiomyocytes	Cardiotoxicity	In vitro	[98]
	Kidney	Nephroprotective	Animal study (rat) and in vitro	[100]
Parecoxib	Heart	Cardio-protective	Animal study (rat)	[99]
Narcotic analgesics				
Morphine	Heart	Cardiotoxicity	Animal study (rat)	[13]
	Liver	Hepatotoxicity	Animal study (rat)	[107]
Tapentadol	Lung, heart, and neurons	Lung, heart, and neuronal toxicities	Animal study (rat)	[110]
	Liver, Kidney	Hepato- and nephrotoxicity	Animal study (rat)	[111]

^1^ NSAIDs; non-steroidal anti-inflammatory drugs, ^2^ COX-II; cyclooxygenase-II.
